# Knee flexion influences periprosthetic BMD measurement in the tibia: Suggestions for a reproducible clinical scan protocol

**DOI:** 10.3109/17453674.2010.501746

**Published:** 2010-07-16

**Authors:** Maiken Stilling, Kjeld Søballe, Kristian Larsen, Niels Trolle Andersen, Ole Rahbek

**Affiliations:** ^1^Department of Orthopaedics, Aarhus University Hospital; ^2^Orthopaedic Research Unit, Hospital Unit West, Holstebro Regional Hospital; ^3^Department of Biostatistics, Aarhus UniversityDenmark

## Abstract

**Background and purpose:**

The quality and quantity of bone is important for the success of joint prostheses and may be monitored by dual energy X-ray absorptiometry (DXA). Available protocols suggest that the knee should be positioned in full extension. This is not possible for most patients in the first days after surgery; however, deficits in extension normalize with rehabilitation. Individual knee flexion between the baseline and follow-up investigations may therefore be different. We investigated the sensitivity of bone mineral density (BMD) measurements to knee flexion in a phantom study and in patients. We suggest a protocol for clinical use.

**Methods:**

2 phantom tibial bones with tibia components were secured in a clamp and BMD measurements were repeated 5 times at every 5° change in flexion from 0° to 20°. For clinical use, a soft foam positioner was produced, in which the lower leg could be placed in neutral rotation and with the knee in approximately 25° of flexion. The clinical repeatability was tested with double examinations in 38 patients. We investigated 3 regions of interest (ROIs) below the tibial plateau.

**Results:**

In the phantom study, just 5° of flexion was found to change the measured mean BMD. The reproducibility of clinical measurements (coefficient of variation) in the 3 ROIs assessed ranged from 1.8% to 3.7% for the anteroposterior scans, and from 3.4% to 6.2% for the lateral scans.

**Interpretation:**

Knee flexion does affect the measured periprosthetic tibial BMD, and knee flexion should be the same at all clinical follow-ups. The protocol and soft foam positioner that we suggest permit precise and reliable assessment of BMD in the proximal tibia and they can be used in clinical work.

## Introduction

The mineral density of trabecular bone is directly associated with both the quality and mechanical properties of the bone, and these are considered to be important predictors of failure in total knee arthroplasty (TKA) (Levitz et al. 1995, Hernandez-Vaquero et al. 2008). Radiostereometric analysis (RSA) is used to measure implant stability as a surrogate marker for the prediction of implant survival (Kärrholm et al. 1994, Ryd et al. 1995), yet in recent years clinical studies have been focusing increasingly on periprosthetic bone (Soininvaara et al. 2004b, Kärrholm and Razaznejad 2008, Digas and Kärrholm 2009). An association between both a low and a high average BMD preoperatively and increased tibial tray subsidence and lift-off has been shown for uncemented implants, although for cemented implants it has been suggested that bone cement can compensate for variations in bone quality in the early period after operation (Li and Nilsson 2000b). Scintigraphic bone assessment of the proximal tibia following cemented TKA has, however, revealed that increased bone remodeling/metabolism continues below the tibial tray for as long as 2 years postoperatively but no concomitant change in bone mineral content was evident in this study (Soininvaara et al. 2008). A number of other studies on periprosthetic bone density after TKA have revealed demineralization of the distal and anterior aspect of the distal femur by up to 36% (Petersen et al. 1995b, Abu-Rajab et al. 2006) and by up to 22% in the proximal tibia (Petersen et al. 1995a), whereas the BMD of the contralateral knee remains unchanged (Karbowski et al. 1999, Soininvaara et al. 2004a). Most reported problems occur with the tibial component (Windsor et al. 1989), and, surprisingly, a high preoperative bone mineral content in the proximal tibia has been associated with later revision surgery (Therbo et al. 2003b). Even so, there is little evidence for a relationship between postoperative densitometric changes after TKA and implant failure (Li and Nilsson 2001, Li et al. 2007).

Dual-energy X-ray absorptiometry (DXA) is a precise and reproducible method for assessment of changes in periprosthetic bone following TKA (Trevisan et al. 1998, Therbo et al. 2003a); however, the precision relies on the quality of the scanner, the quality of the analysis software, and the homogeneity of positioning of the patients at follow-up investigations (Spittlehouse et al. 1999, Li et al. 2004). The use of a heavy-duty polyethylene leg brace to fix the knee in full extension and neutral rotation has been advocated in analysis protocols (Trevisan et al. 1998, Therbo et al. 2003a) and has also been shown to improve the precision of scans in a small scale set-up (Spittlehouse et al. 1999). However, due to pain and swelling, TKA patients often have a temporary extension deficit of the operated knee. Baseline BMD scans are usually performed within the first week after surgery, when many patients may not be able to extend the knee fully, which is often possible in later follow-up scans. The clinical reliability of the fully extended leg position suggested is therefore questionable.

We hypothesized that a change in leg position alone during follow-up of periprosthetic BMD in the tibia would affect the measurements substantially, and we tested the clinical reproducibility of BMD measurements in the proximity of stemmed tibia components with a generally applicable foam positioner that would ensure neutral leg rotation and 25° degrees of flexion.

## Material and methods

### Postoperative flexion deficiency

To establish the clinical importance of knee flexion deficiency during the first week after TKA surgery, we retrospectively evaluated 107 consecutive physiotherapy charts from TKA patients that were filed during a 6-month period in our own department. Quantification of extension deficit was performed as a standard measurement during the postoperative rehabilitation and physiotherapy training. Physiotherapists performed the measurements manually using a protractor with the patient supine on an examination bench.

### Phantom study

**Figure 1.**
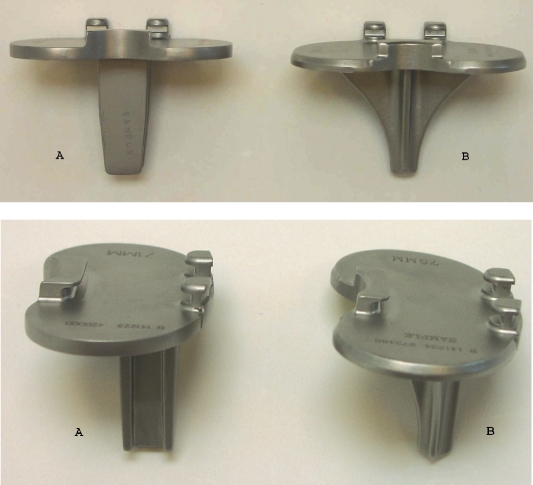
The Maxim I-beam stem (A) and cruxiate (finned) stem (B) tibia stem components used (Biomet).

**Figure 2.**
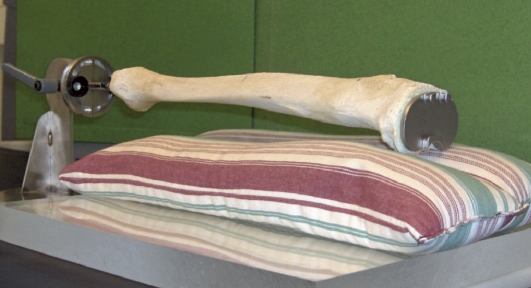
The human phantom bone fixed in neutral flexion on a retrograde nail in a clamp, allowing adjustment of flexion and rotation. Because the scans were made with a “spine program”, we used nylon boards and 2 long rice bags to imitate the soft tissues of the abdomen and loin.

In a phantom study, we investigated the change in measured periprosthetic BMD on anteroposterior (AP) scans with increasing simulated knee flexion. The study was conducted during the last months of 2005 at Aarhus University Hospital, Denmark. 2 tibia-stem designs that were planned to be used in a clinical study were investigated: the Biomet Maxim cruxiate (finned) stem and Biomet Maxim I-beam stem (Figure 1). One I-beam stem and one cruxiate stem tibial component were inserted into right-side dry human phantom tibias and fixed with Palacos bone cement under the implant base plate. 3 regions of interest (ROIs) in the proximal tibia around the tibial components were defined for a planned clinical investigation and the mean value of these regions was assessed. BMD measurements (Lunar Prodigy Advance; General Healthcare, Madison, WI) were repeated 5 times at every 5° of increase in flexion from neutral (0°) to 20° of flexion. The position of the phantom bone was secured in a clamp (Figure 2) that permitted adjustment of flexion with a precision of 1°. As advised by the manufacturer, we used the “spine” scanning mode and used rice and nylon under the tibia as material equivalent to soft tissue.

### In vivo study

**Figure 3.**
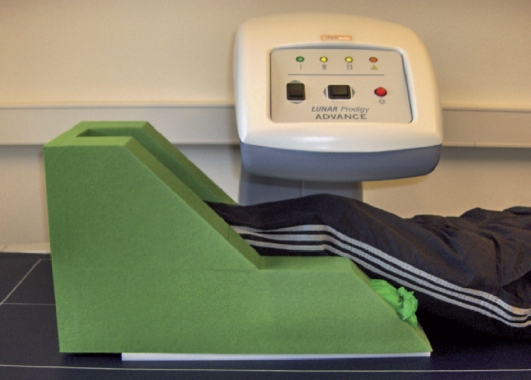
The constructed leg positioner of soft foam for clinical AP scans allowed a reproducible leg position of approximately 25° knee flexion and neutral rotation. The sides of the positioner were filled with rice.

For the clinical study, we designed a generally applicable foam positioner for the leg, to keep the knee semi-flexed by approximately 25° and the lower leg in neutral rotation (toes pointing straight up). The sides of the leg positioner were hollow and filled with rice (Figure 3). We tested the reproducibility of scans in the clinical setting by double BMD measurements using a narrow-angle fan-beam densitometer (Lunar Prodigy Advance; General Healthcare) on 38 patients (mean age 77 (70–85) years, 24 women) at the 6-month follow-up after TKA for osteoarthritis. There were 18 I-beam stem components and 20 cruxiate stem components. Scans were obtained over a period of 2 years during 2006 and 2007 at Aarhus University Hospital, while including patients for a randomized controlled clinical trial. The conditions were everyday working conditions; technicians performed the scans according to a defined protocol. In all subjects, a double AP scan and a lateral (LA) scan were performed by the same scanner as used in the preliminary phantom study, on the same day, and with complete repositioning of the patient. The patients were included in a randomized study approved by the local IRB (issue: 20.01.04; registration: 20030239) and informed consent was obtained from each subject. No intraoperative or long-term complications were registered.

### Scanning and analysis

We used the “AP spine” scan mode in enCORE software version 11.40, with the options “thin” (< 13 cm) and disabling of “smart scan”. The scan window was 18 cm wide and 23.5 cm long as of standards for AP spine mode. The scan was started approximately 25 cm below the inferior patella pole and finished 1–2 sweeps above the proximal part of the tibia component. For AP scans, the leg positioner with rice in the sides was used and the patients were positioned supine. For LA scans, the patient was positioned on the operated side with an approximate 30° of flexion in the hip and knee for comfortable positioning and to keep the tibia parallel to the scanner bed. The knee and lower leg were covered with rice bags for LA scans. The scan resolution was 0.6 × 1.05 mm, average scanning time was 59 seconds, and the average radiation dose was 0.9 mrad (entrance dose).

**Figure 4.**
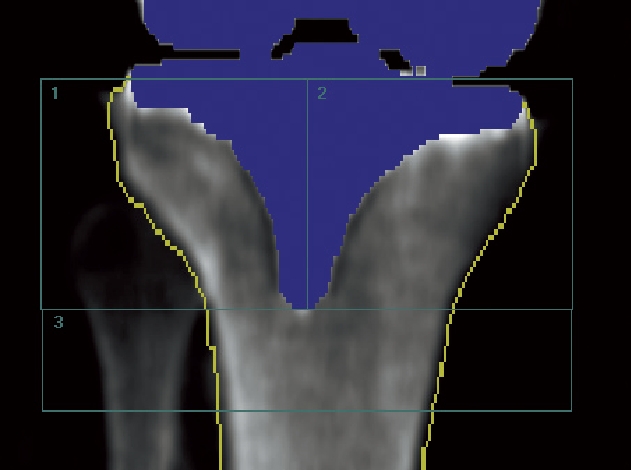
AP densitometry analysis of a right tibia (implant) with software-automated metal removal (blue) and bone-edge detection (yellow line), and manual positioning of the 3-ROI BMD cruxiate stem template. The bone of the fibula was excluded from the analysis.

**Figure 5.**
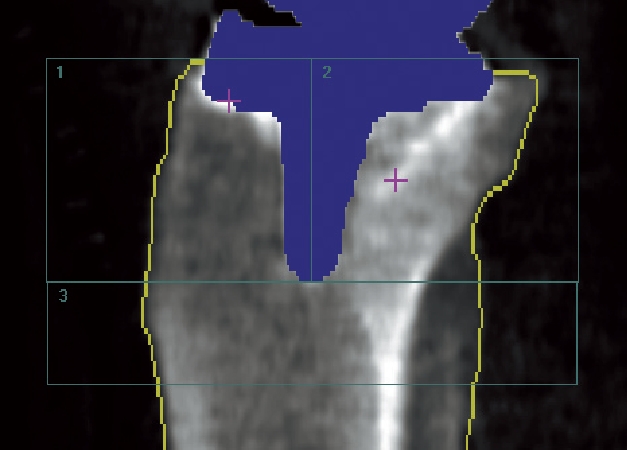
LA densitometry analysis of a right tibia (cruxiate stem implant) with software-automated metal removal (blue) and bone-edge detection (yellow line), and manual positioning of the 3-ROI BMD analysis template. The bone of the fibula was included in the analysis.

We used the ecCORE software for analysis of scan data (phantom and clinical). Using a dynamic tissue detection algorithm in the software, all areas in the scan were point-typed as being bone, tissue, air, metal, or “neutral”. The metal tibia component was thus automatically marked and removed from the densitometry measurement and the edge of the bone was automatically outlined, but manually adjusted when needed. In order to minimize operator-related inaccuracies, we made no attempts to exclude the cement mantle below the tibia base plate from the analysis. A template of 3 ROIs was used. In the AP scans ROI-1 was lateral to the stem and excluded the separable part of the fibula, ROI-2 was medial to the stem, and ROI-3 was inferior to the stem (Figure 4). In the LA scans ROI-1 was anterior to the stem, ROI-2 was posterior to the stem and included the fibula, and ROI-3 was distal to the stem (Figure 5). Once the template of the 3 regions was applied and positioned on the first scan, the template was “locked” to the bone border and could be copied from the baseline scan to subsequent scans, thus facilitating similar positioning of ROIs in follow-up investigations. Manual adjustments of the width of regions were performed to account for individual anatomy. We obtained measurements of BMD (g/cm^2^) for each of the 3 ROIs, and the BMD values of ROI-1 and ROI-2 were switched for left tibias to make all measurements comparable to right tibias. 2 patients had a tissue thickness exceeding the allowed value for thin (< 13 cm) in one of the double AP measurements, which resulted in statistically significantly lower BMD values. Consequently, these scan results were excluded from the statistical analysis, leaving 36 patients for double AP examination analysis and 38 patients for double LA examination analysis.

### Statistics

In the phantom study the mean value of the 5 BMD measurements for each position of flexion, a standard deviation, and the coefficient of variation (CV% = SD/mean × 100) of the method (scanner) was calculated. We used Spearman's rho test to investigate the correlation between flexion and change in BMD. Data within a flexion position were considered to be normally distributed (Shapiro-Wilk test) and we compared the neutral position to increasing degrees of flexion with an unpaired t-test.

For analysis of the clinical repeatability of measurements, we used definitions as described in “ASTM E177-08 for Precision and Bias” (ASTM 2008), where S_r_ is the standard deviation of a single measurement and the 95% repeatability limit is calculated as S_r_ × √2 × 1.96. Bias ± the 95% repeatability limit is identical to the 95% limits of agreement (LOAs) as described by Bland and Altman (1986). The systematic variation (bias) between the double examinations was estimated as the mean difference between the two measurements and presented in Bland-Altman plots for each ROI. The difference between the two measurements followed a normal distribution (Shapiro-Wilk test) and we tested these by a paired t-test. For ease of comparison with the existing literature, the coefficient of variation (CV) of paired measurements was also calculated. Statistical significance was assumed at p < 0.05. Intercooled Stata software version 10.0 (StataCorp, College Station, TX) was used for statistical computations.

## Results

### Postoperative flexion deficiency

68% of 107 random TKA patients in our department had a knee flexion deficiency of 5–30° during the first week of rehabilitation after TKA. Of these 68%, most patients (62%) had a knee flexion deficiency of between 5° and 15° and only a few (6%) had a knee flexion deficiency of between 20° and 30°.

### Phantom study

With increasing flexion, the mean periprosthetic BMD of the phantom bones decreased because the tibial bone was occluded behind the metal shadow of the tibia base plates, and more so for the I-beam stem implant (Spearman's rho = –0.84; p < 0.001) than for the cruxiate stem implant (Spearman's rho = –0.67; p < 0.001). The decrease in measured BMD of the phantom with the cruxiate stem was statistically significant at just 5° of flexion (p < 0.01) compared to neutral position. For the I-beam stem implant, the measured BMD of the phantom decreased at 15° flexion compared to neutral position (p < 0.001) (Table 1). The precision error of the scanner was estimated on the basis of the 5 repetitive scans at each interval of flexion for each phantom bone, and the CV ranged between 0.6% (cruxiate stem implant) and 0.9% (I-beam implant) corresponding to the information from the scanner manufacturer (1–2%).

**Table 1.** Measured periprosthetic BMD (g/cm^2^)) of the phantom bones with increasing degrees of knee flexion. 5 repeat measurements were obtained for each position. A template of 3 ROIs was used. Values are mean (range) and standard deviation (SD)

	Cruxiate stem tibial implant			I-beam stem tibial implant		
	Mean BMD (range)	SD	p-value ^**a**^	Mean BMD (range)	SD	p-value ^**a**^
Neutral	8.465 (8.441–8.515)	0.028		4.735 (4.722–4.730)	0.013	
5° flexion	8.366 (8.321–8.405)	0.032	< 0.01	4.719 (4.706–4.750)	0.019	0.3
10° flexion	8.499 (8.486–8.524)	0.015	0.1	4.711 (4.701–4.749)	0.022	0.2
15° flexion	8.240 (8.119–8.362)	0.096	< 0.01	4.423 (4.374–4.456)	0.035	< 0.01
20° flexion	8.219 (8.166–8.245)	0.031	< 0.01	4.429 (4.299–4.493)	0.075	< 0.01

^**a**^ Statistical testing from neutral to increasing position of flexion (unpaired t-test).

### In vivo study

**Figure 6.**
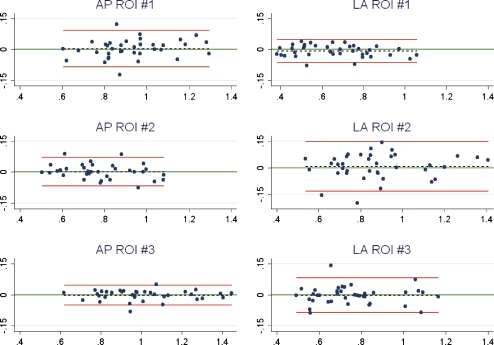
Bland-Altman plot for repeatability (double examinations). X-axis: average of the double measurements; y-axis: difference between double measurements; red lines: 95% limits of agreement; dashed line: bias from 0; long, solid green line: y = 0; dots: individual double values.

Repeatability (precision) of double clinical BMD measurements for the 38 patients had no statistically significant bias (p > 0.1; paired t-test with equal variance) for any ROI, and the 95% repeatability limits (or least significant changes) were small (Table 2 and Figure 6).

**Table 2.** Repeatability of clinical BMD measurements (double examination)

Analysis method	Average BMD ^**a**^ (range)	Bias ^**b**^ (95% CI)	S_r_^**c**^	Repeatability limits ^**d**^
AP measurements				
ROI-1 (g/cm^2^)	0.912 (0.592–1.266)	0.003 (-0.012–0.019)	0.032	0.089
ROI-2 (g/cm^2^)	0.793 (0.503–1.080)	0.002 (-0.010–0.014)	0.025	0.070
ROI-3 (g/cm^2^)	1.010 (0.617–1.446)	-0.002 (-0.010–0.006)	0.017	0.048
LA measurements				
ROI-1 (g/cm^2^)	0.659 (0.383–1.056)	-0.007 (-0.016–0.002)	0.020	0.055
ROI-2 (g/cm^2^)	0.882 (0.538–1.408)	0.007 (-0.015–0.030)	0.050	0.140
ROI-3 (g/cm^2^)	0.757 (0.492–1.165)	-0.002 (-0.016–0.011)	0.030	0.084

^**a**^ Average of the mean of double BMD measurements with the range in parentheses.

^**b**^ Bias: mean difference between the first and the second BMD measurement (systematic variation of repeatability within the ROI).

^**c**^ S_r_: repeatability standard deviation for a single BMD measurement (ASTM 2008).

^**d**^ 95% limit repeatability of 2 test results (1.96 × √2 × S_r_).

The CVs of paired measurements on AP scans were 3.7%, 3.2%, and 1.8% for measurements in ROI-1, ROI-2, and ROI-3. The CVs of paired measurements on LA scans were 3.4%, 6.2%, and 4.3% for measurements in ROI-1, ROI-2, and ROI-3.

## Discussion

To our knowledge, there have been no previous studies exploring the sensitivity of knee flexion to changes in periprosthetic BMD in TKA. We found that flexion deficiency (range 5–30°) is a problem for two-thirds of patients in the first days after TKA surgery, and that even small changes in knee flexion (range 5–15°) substantially influence the periprosthetic bone density measured in the proximal tibia. Our study shows that a protocol controlling knee flexion results in reproducible data when measuring the periprosthetic BMD of the tibia component of cemented TKA.

In clinical investigations, the baseline DXA scan of the periprosthetic tibia bone is best performed within a few days after surgery to match the timing of other postoperative data, e.g. RSA (Valstar et al. 2005). The reported prevalence of knee stiffness with a flexion deficiency of more than 15° after 1.5 years following TKA is only 1% (Kim et al. 2004), which means that most patients are likely to position the knee differently at the baseline and the follow-up AP DXA scans. Our phantom investigation confirmed that a change in knee flexion between densitometry scans changes the measured periprosthetic BMD, and at smaller degrees of flexion for a cruxiate (finned) stem tibia component (5°) than for a straight stem (I-beam) tibia component (15°) due to the shadowing of bone just beneath the tibia base plate by the finned stem, and to a lesser degree shadowing in the diaphyseal ROI by the tip of the stem. To overcome this problem, we suggest using a clinical AP densitometry scan protocol featuring a soft foam leg positioner (Figure 3) to hold the lower leg in neutral rotation and the knee in approximately 25° of flexion at all scans. This is a usable set-up both postoperatively and at later follow-ups, and our double clinical measurements have confirmed that there is a high degree of precision with CVs between 1.8% and 3.7% for the most and least precisely assessed ROI (AP scans); this is in accordance with or slightly better than in earlier reports (Trevisan et al. 1998, Soininvaara et al. 2004b). For the LA scans, the patients were simply positioned on the operated side with the knee semi-flexed and the tibia parallel to the scanner bed. We found that precision based on double clinical measurements was also satisfactory for LA scans, although lower than for AP scans, with CVs between 3.4% and 6.2% for the most precise and least precise region. As has also been formerly documented (Therbo et al. 2003a), we obtained the best precision for BMD measurements in AP scans of ROI-3, which was below the stem and free of the implant metal. For the LA scans the best precision was in ROI-1, which is anterior to the stem and which was free of problems of varying occlusion of the fibula. In agreement with this, there was most variation (wider agreement limits) in LA ROI -2 and AP ROI-1 where the fibula is located (Figure 6).

Assessment of individual changes in periprosthetic BMD in prospective clinical studies necessitates the use of a method with high precision (low error), and all clinical studies should report the clinical precision of the method used based on double measurements (Malchau 2000). Clinically meaningful changes in BMD were much higher than the imprecision of our DXA scanner, which permits assessment of small sample sizes and/or small regions of interest in clinical follow-up investigations (Cohen and Rushton 1995). Currently, activities at the bone-implant interface (requiring small ROIs and high precision) are of interest for establishment of any association between implant migration/failure and periprosthetic BMD change (Li and Nilsson 2001). We calculated the least significant change (LSC) in BMD as the 95% repeatability limits (ASTM 2008) for the 38 patients, and our results suggest that a relative BMD change in AP ROIs of between 5% (ROI-3) and 10% (ROI-1) would be detectable. For the LA ROIs, a relative BMD change of between 8% (ROI-1) and 16% (ROI-2) would be detectable. Furthermore, an absolute LSC between 0.048 g/cm^2^ (ROI-3) and 0.089 g/cm^2^ (ROI-1) for the AP scans, and a change between 0.055 g/cm^2^ (ROI-1) and 0.140 g/cm^2^ (ROI-2) for the LA scans would be detectable. The International Society for Clinical Densitometry (ISCD) provides a calculating tool for easy assessment of precision (the LSC at the 95% confidence level) for either 15 or 30 patients. This tool calculates “the 95% repeatability limits” and may be used as a preliminary tool for estimation of the precision of measurements during data collection for a clinical trial. However, final precision should be based on double measurement of all patients investigated.

Previous densitometry studies on the proximal tibia have reported periprosthetic decreases in BMD of 5% at 6 months (Spittlehouse et al. 1999), 10% at 1 year (Karbowski et al. 1999), 13% after 3 months (Li and Nilsson 2000a), 20% at 6 months (Petersen et al. 1995a), and 26% (medial tibial condyle) after 5 years (Regner et al. 1999). It has been shown that the precision of osteoporosis scans (spine, total hip, and femoral neck) is approximately twice as poor for long-term follow-ups than for short-term follow-ups (Tothill and Hannan 2007), and this should be kept in mind for other anatomical regions when examining progressive bone changes, because a poorer long-term precision could lead to an underestimate of actual changes in bone density. In comparison, for measurement of BMD in the spine and hip, a 2-fold reduction of precision at long-term follow-up as compared to short-term follow-up leads to an underestimate of change in BMD in 25% of patients (Tothill and Hannan 2007).

Software-associated difficulties with automated bone and implant identification necessitate manual analysis in some cases, which inevitably leads to a reduction in precision (Spittlehouse et al. 1999). Anatomical variation makes the fibula more or less separable from the tibia, especially on the LA scans, and also frequently necessitates manual override in the analysis. When using computer programs that are developed for different anatomical regions, it is necessary to imitate the expected tissue-equivalent density by use of tissue-equivalent material. Rice, nylon, or water bags are commonly used to trick the software into running in automatic mode and to avoid air gaps when these are not expected by the software. We used a “spine program” and thus had to mimic the anatomical region of the “stomach and loin” by accounting for tissue thickness (rice) and scan area, and we had to avoid air around the knee (personal communication Lunar; GE Healthcare). The rice was incorporated in the sides of the positioner; thus, we did not have any problem re-establishing the same “tissue-thickness” from scan to scan. However, the positioner was quite heavy and not optimal for routine clinical use. A “knee program” has been developed by the manufacturer since we performed this study. The knee program alleviates the use of tissue aids and makes clinical use much simpler; however, it is currently only available for use in clinical research and its precision for periprosthetic BMD measurement in the tibia has not been evaluated.

The size and placement of regions of interest in the proximal tibia have varied between studies but commonly, ROIs are applied medial and lateral to the stem and anterior and posterior to the stem (Trevisan et al. 1998, Soininvaara et al. 2004a, Abu-Rajab et al. 2006). Different programs may permit easy application of ROI templates to successive follow-up scans by locking the template to the bone contour, as in our case, or impose a less precise manual positioning of ROIs (Li et al. 2004). In our experience, small adjustments in point-typing of tissue (bone and metal artifact) only improve precision slightly while a homogenous leg position is of greater importance for high precision and reliable results. This is because in contrast to radiostereometric analysis, for example, DXA does not offer any basic calibration system to correct for slight changes in patient position. In the absence of a calibration system for DXA set-up, the ideal solution for maintainenance of high precision by identical patient positioning is a patient-specific positioner similar to those used for radiation treatment in oncology. In most departments, this is not a practical solution for financial reasons and because of limited space for storage. A few different brace sizes with standard flexion would make it possible to store the devices.

A limitation of this study was that we only investigated the effect of flexion on 2 phantom bones (1 with a tibia component with a cruxiate (finned) stem and 1 tibia component with a straight (I-beam) stem). However, if significant changes in BMD measurements due to leg positioning can be demonstrated with 2 phantom bones, then the same problem would be likely to occur and affect the results of at least some measurements in a randomized clinical study. The strength of our clinical study (with double measurements) is that it included many patients, and that the generalizability of the results was high because scans were obtained as is standard in a clinical study, with a long follow-up time (2 years) and performed by several technicians according to a predefined protocol. The precision shown is thus reliable and the results should be reproducible in any institution with similar equipment.

In conclusion, a clinically applicable soft foam positioner designed to ensure rotational stability and to allow for slight flexion (i.e. 25°) is safe for clinical use, because this position can be obtained with all normal TKA patients both in the early period after surgery and in later follow-ups. However, even with a leg positioner at hand, a dedicated protocol must be available and the positioning of the lower leg and knee must be handled meticulously to obtain high-precision scans over a long period of time by several technicians, which are the typical conditions in clinical studies. For each patient, we recommend that the radiographic appearance of the extremity should be confirmed with previously obtained scans to make sure that the position of the extremity similar on the follow-up scan.
